# Dedicated AI Expert System vs Generative AI With Large Language Model for Clinical Diagnoses

**DOI:** 10.1001/jamanetworkopen.2025.12994

**Published:** 2025-05-29

**Authors:** Mitchell J. Feldman, Edward P. Hoffer, Jared J. Conley, Jaime Chang, Jeanhee A. Chung, Michael C. Jernigan, William T. Lester, Zachary H. Strasser, Henry C. Chueh

**Affiliations:** 1 Laboratory of Computer Science, Massachusetts General Hospital, Boston

## Abstract

**Importance:**

Large language models (LLMs) have not yet been compared with traditional diagnostic decision support systems (DDSSs) on unpublished clinical cases.

**Objective:**

To compare the performance of 2 widely used LLMs (ChatGPT, version 4 [hereafter, *LLM1*] and Gemini, version 1.5 [hereafter, *LLM2*]) with a DDSS (DXplain [hereafter, *DDSS*]) on 36 unpublished general medicine cases.

**Design, Setting, and Participants:**

This diagnostic study, conducted from October 6, 2023, to November 22, 2024, looked for the presence of the known case diagnosis in the differential diagnoses of the LLMs and DDSS after data from previously unpublished clinical cases from 3 academic medical centers were entered. The systems’ performance was assessed both with and without laboratory test data. Each case was reviewed by 3 physicians blinded to the case diagnosis. Physicians identified all clinical findings as well as the subset deemed relevant to making the diagnosis for mapping to the DDSS’s controlled vocabulary. Two other physicians, also blinded to the diagnoses, entered the data from these cases into the DDSS, LLM1, and LLM2.

**Exposures:**

All cases were entered into each LLM twice, with and without laboratory test results. For the DDSS, each case was entered 4 times: for all findings and for findings relevant to the diagnosis, each with and without laboratory test results. The top 25 diagnoses in each resulting differential diagnosis were reviewed.

**Main Outcomes and Measures:**

Presence or absence of the case diagnosis in the system’s differential diagnosis and, when present, in which quintile it appeared in the top 25 diagnoses.

**Results:**

Among 36 patient cases of various races and ethnicities, genders, and ages (mean [SD] age, 51.4 [16.4] years), in the version with all findings but no laboratory test results, the DDSS listed the case diagnosis in its differential diagnosis more often (56% [20 of 36]) than LLM1 (42% [15 of 36]) and LLM2 (39% [14 of 36]), although this difference did not reach statistical significance (DDSS vs LLMI, *P* = .09; DDSS vs LLM2, *P* = .08). All 3 systems listed the case diagnosis in most cases if laboratory test results were included (all findings DDSS, 72% [26 of 36]; LLM1, 64% [23 of 36]; and LLM2, 58% [21 of 36]).

**Conclusions and Relevance:**

In this diagnostic study comparing the performance of a traditional DDSS and current LLMs on unpublished clinical cases, in most cases, every system listed the case diagnosis in their top 25 diagnoses if laboratory test results were included. A hybrid approach that combines the parsing and expository linguistic capabilities of LLMs with the deterministic and explanatory capabilities of traditional DDSSs may produce synergistic benefits.

## Key Points

**Question:**

How does the performance of a dedicated artificial intelligence (AI) expert system for clinical diagnosis compare with that of 2 generative AI large language models (LLMs)?

**Findings:**

In this diagnostic study, the dedicated AI diagnostic decision support system listed the correct diagnosis more often, and higher up, in its differential diagnosis than the LLMs, but the LLMs also performed well.

**Meaning:**

This study suggests that more than 1 type of decision support tool can provide helpful diagnostic assistance to clinicians.

## Teaser

This diagnostic study compares the performance of 2 widely used large language models with an artificial intelligence diagnostic decision support system on 36 unpublished general medicine cases.

## Introduction

The use of artificial intelligence (AI) in medicine goes back many decades,^1,2^ but interest has increased markedly since generative AI based on large language models (LLMs) emerged. Large language models have been reported to do as well as physicians in passing many board examinations, although not in all disciplines.^3,4^ Large language models have also been tested for their ability to analyze case descriptions and generate accurate diagnoses, and have performed well in some cases, but did not reach the accuracy of physicians in others.^5,6^ These results are noteworthy, as generative AI was not designed for clinical reasoning but generates human-like text responses to any question using enormous datasets gathered from the internet.

Traditional AI expert systems as medical diagnostic decision support systems (DDSSs) have evolved over 4 decades.^7,8^ DXplain (January 7, 2024, through May 22, 2024; Massachusetts General Hospital [MGH]; hereafter, *the DDSS*) is one of these, developed at the MGH Laboratory of Computer Science in the 1980s as a stand-alone program, evolving into a web-based application and cloud-based differential diagnosis engine. The DDSS’s knowledge base contains more than 2680 disease profiles, more than 6100 clinical findings (history, physical examination, and laboratory terms), and hundreds of thousands of data points that describe the relationships between them. A user can enter clinical findings and the DDSS will generate a rank-ordered list of diagnoses that explain the findings. The DDSS has been shown to improve the accuracy of medical residents’ diagnostic abilities, shorten the length of stay of medical inpatients with complex conditions, and reveal findings with high predictive value for critical diseases that could allow for their earlier detection.^9,10,11,12^

Given the interest in generative AI and LLMs for medicine, we undertook a comparison of the diagnostic accuracy of the DDSS with that of 2 widely used LLMs: ChatGPT (Chat Generative Pre-trained Transformer), version 4 (March 20-23, 2024; Open AI [hereafter, *LLM1*]) and Gemini, version 1.5 (March 20, 2024, and March 28, 2024; Google [hereafter, *LLM2*]). These 2 LLMs performed best when 5 LLMs were used to analyze *New England Journal of Medicine* and *JAMA* clinical challenge cases.^13^

## Methods

This diagnostic study was conducted from October 6, 2023, to November 22, 2024. The MGH institutional review board determined that this study did not meet criteria for human participants research and therefore did not require institutional review board approval. We followed the Standards for Reporting of Diagnostic Accuracy (STARD) reporting guideline for diagnostic studies.^14^

A collection of 36 clinical cases based on actual patients developed by 3 academic medical centers was used in this study. The cases are known to be diagnostically challenging, with final diagnoses based on definitive tests, clinical follow-up, or autopsy.^15^ The case authors redacted any information that was considered definitive or that would have made the diagnosis obvious or trivial. Provided to us by their developers over 20 years ago, these cases, to our knowledge, have not been previously published, nor did internet searches or generative AI queries reveal that they were. We have never used these cases in the construction of the DDSS algorithm or knowledge base. Hence, we considered all systems we tested to be naive to these specific cases.

Unlike generative AI systems that accept narrative text, the DDSS requires a user to enter findings from a controlled vocabulary by searching its dictionary, assisted by its use of common lexical techniques such as key word matching with stemming. Consequently, for the purposes of this study, individual findings from each case had to be extracted and then mapped to the DDSS’s clinical vocabulary. For the extraction task, 6 clinicians were recruited (J.J.C., J.C., J.A.C, M.C.J., W.T.L., and Z.H.S.) who were not part of the DDSS team and who had no previous experience with these cases. All are board-certified internists or emergency medicine physicians on staff at MGH. Each physician received a set of 18 case narratives (without diagnoses) and was asked to return a marked-up copy identifying all clinical findings, as well as only those findings thought to be relevant for establishing diagnoses (positive and negative findings for both). With this approach, every individual case was reviewed by 3 different physicians.

For the mapping task, 2 of us (M.J.F. and E.P.H.), also blinded to the case diagnoses, consolidated the results from the previous step prior to entering cases into the DDSS and LLMs. For each case, only clinical findings identified by at least 2 of 3 physicians were selected to be in the “all clinical findings” or the “findings relevant for making a diagnosis” collections. We then mapped each of these findings to the DDSS vocabulary. For quantitative laboratory test results, the DDSS represents only relative laboratory test values, such as high or normal. To avoid skewing the results with trivial laboratory test abnormalities, we considered values within 5% of the limits of the reference range as normal.

Four variations of each case were input into the DDSS: all clinical findings (DDSS ALL) (1) including and (2) excluding laboratory test results and only findings relevant to making a diagnosis (DDSS REL) (3) with and (4) without laboratory test results. For both LLMs, 2 variations of the cases were entered: (1) the complete text of the case including laboratory test data and (2) only the narrative portion of the case excluding laboratory test data.

The initial prompt used for the LLMs was: “Act as if you were the discussant at a hospital conference. Given the following scenario, what would be the diagnoses you would consider, in rank order from most likely to least likely. Please list at least 25 diagnoses. Please be as specific as you can when listing diagnoses.”

Halfway through its cases, LLM2 displayed that “it was only a large language model and could not provide a response.” The first sentence of the above prompt for LLM2 was replaced by “To the best of your ability as a language model,” at which point it complied.

The goal of this study was to evaluate whether the case diagnosis was listed in the DDSS’s or the LLMs’ differential diagnosis and at what rank. The overall quality of the entire differential diagnosis was not evaluated. We compared the DDSS’s top 25 diagnoses with the 25 diagnoses generated by each LLM. In deciding whether a diagnostic label provided by the DDSS or either of the LLMs correctly matched the case diagnosis provided by the case authors, we used a comprehensive compilation provided by the case authors that lists all acceptable synonyms for case diagnoses.

### Statistical Analysis

The output from each LLM for each of the 36 cases was compared with both versions of the DDSS’s output: DDSS REL and DDSS ALL. This was done first using cases without laboratory test results and then repeated using cases with laboratory test results. Hence, there were 4 comparisons between the DDSS and each LLM: the DDSS REL and the DDSS ALL, each with and without laboratory test results.

We analyzed the data using a quintile strategy: 5 points were awarded if the correct diagnosis was ranked from first to fifth in the differential diagnosis, 4 points if ranked from sixth to tenth diagnosis, and so on down to 1 point if the correct diagnosis was ranked from 21st to 25th. Diagnoses ranked lower than 25 were treated as unlisted (no points awarded).

Quintile scores calculated for DDSS ALL, LLM1, and LLM2 were compared using mixed model analysis of variance for repeated measures. The frequencies with which the case diagnosis was listed within the top quintile of diagnoses made by DDSS ALL, LLM1, and LLM2 were compared using a logit-link generalized linear model for which the estimation was carried out using the generalized estimating equations approach. The Fisher exact test was applied to compare the proportions of correct diagnoses among DDSS ALL, LLM1, and LLM2. All *P* values were from 2-sided tests, and results were deemed statistically significant at *P* < .05 (SAS, version 9.4; SAS Institute Inc).

## Results

Thirty-six patient cases of various races and ethnicities, genders, and ages (mean [SD] age, 51.4 [16.4] years) were used. In a representative subset of cases, a mean (SD) of 20% (15%) of case findings were identified as findings thought likely to be relevant for establishing diagnoses by all 3 physician reviewers, a mean (SD) of 30% (13%) were identified by 2 of 3 physician reviewers, and a mean (SD) of 50% (1%) were identified by 1 of 3 physician reviewers. For the all clinical findings category, a mean (SD) of 91% (2%) of findings were identified by all 3 physician reviewers, a mean (SD) of 8% (2%) were identified by 2 of 3 physician reviewers, and a mean (SD) of 1% (1%) were identified by 1 of 3 physician reviewers. Findings thought to be relevant is a subset of all clinical findings.

Scoring results are included in graphic and tabular form in aggregate for the 36 cases using the quintile strategy scoring system. They show that the case diagnosis appeared higher on the DDSS’s ranked list more often than on each of the LLMs’ lists in all 4 comparisons: laboratory test results excluded or included and all or only relevant findings provided to the DDSS (Figure). In the all findings without laboratory test results version, the DDSS listed the case diagnosis in its differential diagnosis more often (56% [20 of 36]) than LLM1 (42% [15 of 36]) and LLM2 (39% [14 of 36]), although this difference did not reach statistical significance (DDSS vs LLMI, *P* = .09; DDSS vs LLM2, *P* = .08). All 3 systems listed the case diagnosis in most cases if laboratory test results were included (all findings DDSS, 72% [26 of 36]; LLM1, 64% [23 of 36]; and LLM2, 58% [21 of 36]) (Table). In addition, the Table shows that case diagnoses generally appeared at least somewhere among the DDSS’s differential diagnoses, and also within its top 5, more often than among the LLMs’ differential diagnoses. Although there was considerable overlap, there was some notable variability. In at least 1 of the 4 versions (all results, relevant results, with or without laboratory test results), the correct diagnosis appeared on the DDSS’s list in 27 of the 36 cases, on LLM1’s list in 24 of the 36 and on LLM2’s list in 22 of 36 cases. Statistical significance was not reached in any of the comparisons, but trends favored DDSS ALL without laboratory test results and the DDSS listing the correct diagnosis higher in its differential diagnosis compared with LLM2 than with LLM1 (Figure).

**Figure.**
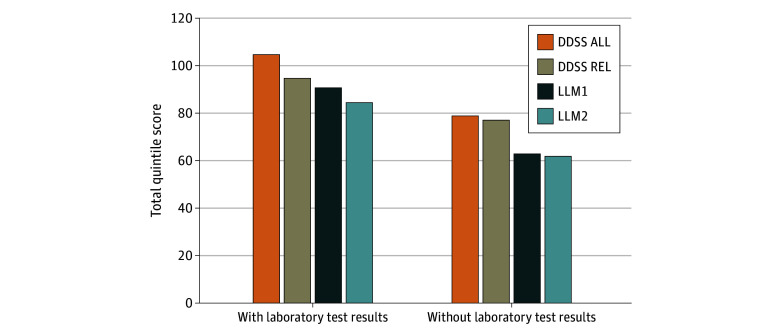
Diagnostic Decision Support System (DDSS) vs Large Language Models (LLMs) Comparison of performance across systems for placing the correct diagnosis higher up on a differential diagnosis consisting of 25 diagnoses. DDSS ALL indicates the DDSS with all clinical data; DDSS REL, the DDSS with only clinical data considered relevant.

**Table.** Comparison of Performance Across Systems on 36 Cases for Placing the Correct Diagnosis Higher on a Differential Diagnosis Consisting of 25 Diagnoses

Characteristic	No laboratory test results				With laboratory test results			
	LLM1	LLM2	DDSS ALL	DDSS REL	LLM1	LLM2	DDSS ALL	DDSS REL
Quintile total score^a^	63	62	79	77	91	85	105	95
*P* value^b^	.40	.37	NA	NA	.45	.28	NA	NA
Quintile score, mean (SD)	1.8 (2.2)	1.7 (2.2)	2.2 (2.2)	2.1 (2.2)	2.5 (2.2)	2.4 (2.2)	2.9 (2.1)	2.6 (2.2)
Diagnosis anywhere in DDX, No./total No. (%)	15/36 (42)	14/36 (39)	20/36 (56)	18/36 (50)	23/36 (64)	21/36 (58)	26/36 (72)	23/36 (64)
*P* value^b^	.09	.08	NA	NA	.36	.19	NA	NA
Diagnosis in top quintile, No./total No. (%)	7/36 (19)	10/36 (28)	10/36 (28)	10/36 (28)	13/36 (36)	8/36 (22)	14/36 (39)	14/36 (39)
*P* value^b^	.25	>.99	NA	NA	.76	.11	NA	NA

Abbreviations: ALL, all clinical findings; DDSS, diagnostic decision support system; DDX, rank-ordered differential diagnosis list of 25 diagnoses; LLM, large language model; NA, not applicable; REL, findings relevant for arriving at the patient’s diagnosis.

^a^ Quintile scoring method: 5 points awarded for diagnosis ranked 1 to 5, 4 points awarded for diagnosis ranked 6 to 10, and so on down to 1 point awarded for diagnosis ranked 21 to 25.

^b^ *P* value reflects comparison between the DDSS with all findings vs LLMs and therefore is listed only in LLM columns.

The case diagnosis was found on the DDSS’s list in 7 of the 12 cases (58%) that LLM1 missed and in 9 of the 14 cases (64%) that LLM2 missed (Table). However, the case diagnosis appeared on both LLM1’s and LLM2’s lists in 4 of the 9 cases (44%) that the DDSS missed: 6 cases in total that the LLMs listed correctly that the DDSS did not; 2 cases were common to both LLMs, and each LLM listed an additional 2 diagnoses that neither the DDSS nor the other LLM listed.

We also analyzed the data using a “winner take all” approach, where a single point was awarded to whichever entity (the DDSS or the LLM) ranked the correct diagnosis higher on its list. The DDSS achieved higher scores than the LLMs, although, again, this did not reach statistical significance (eAppendix in Supplement 1).

## Discussion

Amid all the interest in LLMs, it is easy to forget that the first AI systems used successfully in medicine were expert systems. These systems, including the DDSS, use the computer’s ability to store and retrieve enormous amounts of data consistently to perform as would an expert with perfect memory and inexhaustible energy. The DDSS may enhance and expand clinicians’ differential diagnoses because it can recall information that humans may forget in the heat of the moment and it is not biased by common flaws in human diagnostic reasoning, such as anchoring or recency bias. Generative AI models have quickly produced similar levels of diagnostic performance as expert systems.

We chose the 2 versions of case entry for the DDSS (DDSS ALL, DDSS REL) because the former is how a future automated electronic health record–integrated approach would likely be implemented and the latter is how the system is currently used in practice. The DDSS knowledge base contained all 36 of the correct case diagnoses. Of the 9 cases where the DDSS did not list the case diagnosis in its top 25, reasons included the following:

- Several cases consisted of many nonspecific findings (eg, fever, anemia, thrombocytopenia, chills), and the case diagnosis was a rare disease. When many diagnoses are equally (and usually weakly) supported by nonspecific findings, the DDSS prioritizes common diseases over rare ones, as it uses quasiprobabilistic algorithms. Some variations of the DDSS user interface highlight the top rare diseases separately, which mitigates this issue.
- Missing information in the DDSS knowledge base (eg, the finding “IgM [immunoglobulin M] kappa monoclonal protein” was missing from the disease profile of cryoglobulinemia).
- Insufficient, contradictory data or both. The case description of subarachnoid hemorrhage (SAH) included no classic findings, an atypically long 2-week presentation, and the notation “head computed tomography showed no SAH.”
- Incorrect information in the DDSS knowledge base: In a case of aortic dissection, the patient had a 3-week duration of chest pain. The DDSS placed too great of a negative weight for a prolonged presentation of this condition, depressing its ranking.

As expected, both the DDSS and the LLMs performed better when laboratory test results were included (Figure). The case diagnosis appeared more often and slightly higher on the DDSS’s differential diagnosis than the LLMs’, although without statistical significance. The LLMs performed remarkably well considering they were not designed for the medical domain. Also shown is the variation in the DDSS’s performance between all findings and relevant findings versions compared with the LLMs’ performance, both with and without laboratory test results. When laboratory test results were not entered, there was minimal difference between the all findings and relevant findings versions. However, when laboratory test results were included, the DDSS’s performance was better with all findings. This suggests that a DDSS that captures all laboratory test results is preferable to one where only selected laboratory test results are available. Hence, integration with the clinical workflow where all data are available should allow for improved performance when compared with the current method of clinician case entry of selected findings ex post facto.

Most other studies that have evaluated LLMs’ diagnostic capabilities on challenging cases included cases available on the internet; hence, LLMs may have been trained on those data. The cases we used were never published, so this potential problem is avoided.^13,16,17,18^ In a recent report, Rutledge^19^ used the same 36 cases used in our study and found that LLM1 included the correct diagnosis in its top 6 diagnoses 61% of the time. This finding correlates well with our observation that LLM1 included the case diagnosis in 64% of cases (23 of 36), with the difference partially explainable by our including the presence of the correct diagnosis in the top 25 diagnoses.

The beneficial features of LLMs include widespread availability, capacity to accept large amounts of narrative text with little to no physician input, ability to produce highly readable output, and ability to be updated using automation rather than manual curation. The most negative characteristics include the lack of repeatability (different responses can be generated with the same data input), the lack of reliability (generative AI can “confabulate” or “hallucinate” and craft responses with entirely false facts), and the lack of an explanation (although increasingly the latest LLMs are annotating responses with references). Confabulation or hallucination imply an element of volition or consciousness that cannot yet be ascribed to LLMs at the level of human capability. Confabulation might be better termed an algorithmic shortcoming due to probabilistic adjacency.

Physicians are more likely to accept the diagnostic suggestions of a medical DDSS if the system can explain its reasoning. Large language models are predominantly black boxes (ie, systems that can be viewed in terms of their inputs and outputs, with no knowledge of their internal workings) and typically fail to provide the reasoning behind their outputs. In contrast, the DDSS was designed inherently to be transparent in supporting its conclusions. The term *DXplain* is a portmanteau of *DX* (diagnosis) and *explain*. By clicking on any diagnosis in the DDSS’s list, users can access explanations of the clinical findings supporting that diagnosis along with additional findings that would further substantiate it.

The issue of black box behavior is a foundational problem in AI, extending from modern AI systems such as LLMs back to the roots of AI with perceptrons and artificial neural systems. As Minsky and Papert^20^ highlighted in their seminal 1969 book, *Perceptrons: An Introduction to Computational Geometry*, this inherent opacity limits trust—a challenge that persists in contemporary AI models, including LLMs—that is eroded further by the propensity for confabulation. Ultimately, the lack of trustworthiness is one of the major barriers to the implementation of these systems.

The DDSS listed the case diagnosis in its differential diagnosis more than half the time that either LLM did not include it (58% LLM1, 64% LLM2), and each LLM listed the case diagnosis 44% of the time that the DDSS did not list it. A hybrid approach using both types of tools could improve both systems. For example, querying the LLMs to support their reasoning for including the correct diagnoses that the DDSS missed could help the developers correct any knowledge base errors. Conversely, asking an LLM to consider a diagnosis that the DDSS listed that the LLM did not list might allow the LLM to reconsider its differential diagnosis.

## Limitations

This study has some limitations. The versions of LLMs used in this study were current as of the time of manuscript submission. These findings are likely to change as LLMs rapidly evolve. Looking at the variation in performance between expert systems and generative AI models can reveal some relevant insights for the design of future systems. We evaluated only 1 DDSS, and there are other medical DDSS tools available for clinical use. The result of 1 study evaluating 4 medical DDSSs including DXplain found DXplain’s performance to be among the highest.^21^ We sought only the presence of the case diagnosis rather than an evaluation of the quality of the differential diagnosis. In the original study that used these 36 cases,^15^ the authors argue that the true benefit of a diagnostic decision support system is whether it enhances clinician performance (ie, the performance of a clinician aided by a decision support system is superior to that of a clinician alone). As such, their study evaluated 2 different decision support systems using these 36 cases with medical students, residents, and attending physicians. It showed a benefit, with decision support system consultation improving the presence of the diagnosis as well as the quality of the differential diagnosis when compared with pre–decision support system consultation, with medical students improving the most. Given the novelty of LLMs, we chose to evaluate whether the correct diagnosis was contained in their differential diagnoses. We view this as a foundational prerequisite to a future study that could evaluate the performance of a clinician using an LLM vs a clinician alone. If the case diagnosis is not included in the decision support system’s differential diagnosis, then the decision support system is less likely to be helpful to the user.

## Conclusions

We have found that existing LLM and natural language processing tools excel at extracting clinical findings from medical record prose. They can also be used to augment existing systems; for example, we have done preliminary work that suggests that an LLM can be used to reliably extract and map clinical findings from narrative text for input into the DDSS. We are excited about combining the new generation of AI tools with existing systems to better automate diagnostic decision support because even the most effective tool would not be beneficial if it is not used. Just as drug interaction software can alert a clinician if 2 or more drugs ordered for a patient have harmful interactions, so too might a tool be able to alert the clinician if there is sufficient support for a diagnosis that has not yet been considered (eg, not on the patient’s problem list). Given that the DDSS failed to list 9 diagnoses in a ranking of 25, yet 6 of these were listed by either one or both LLMs, this suggests the possibility that a hybrid approach using the LLMs to augment the decision support capabilities of medical DDSS holds promise.

## Notes

Supplement 1. **eAppendix.** Data for “Winner Take All” (WTA) Approach

Supplement 2.
Data Sharing Statement
