# Supplementary material for: Dedicated AI Expert System vs Generative AI With Large Language Model for Clinical Diagnoses
Source: JAMA Netw Open. 2025 May 29;8(5):e2512994. doi: 10.1001/jamanetworkopen.2025.12994 (PMC12123466; doi:10.1001/jamanetworkopen.2025.12994)
Supplement: Supplement 1. — eAppendix. Data for “Winner Take All” (WTA) Approach [file jamanetwopen-e2512994-s001.pdf]

Supplementary Online Content

Feldman MJ, Hoffer EP, Conley JJ, et al. Dedicated AI expert system and generative AI with large language model for clinical diagnoses. *JAMA Netw Open.* 2025;8(5):e2512994. doi:10.1001/jamanetworkopen.2025.12994

eAppendix. Data for "Winner Take All" (WTA) Approach

This supplementary material has been provided by the authors to give readers additional information about their work.

© 2025 Feldman MJ et al. *JAMA Network Open.*

eAppendix. Data for "Winner Take All" (WTA) Approach

A single point was awarded to whichever entity (DXplain or the LLM) ranked the correct diagnosis higher on its list. DXplain achieved higher scores though this did not reach statistical significance. Totals do not add to 36 (total number of cases) because points were not awarded if neither tool had the case diagnosis on its list.

Chat GPT no labs	DXp REL no labs
WTA	WTA
12 (0.82)	13

Gemini no labs	DXp REL no labs
WTA	WTA
11 (0.81)	12

Chat GPT with labs	DXp REL with labs
WTA	WTA
14 (0.51)	17

Gemini with labs	DXp REL with labs
WTA	WTA
15 (0.68)	17

Chat GPT no labs	DXp ALL no labs
WTA	WTA
12 (0.64)	14

Gemini no labs	DXp ALL no labs
WTA	WTA
9 (0.13)	16

Chat GPT with labs	DXp ALL with labs
WTA	WTA
15 (0.43)	19

Gemini with labs	DXp ALL with labs
WTA	WTA
13 (0.19)	20

Pair-wise comparisons between DXplain and LLM with and without labs

Score (p-value); p-value listed only in LLM column
REL: findings relevant for arriving at the patient's diagnosis
ALL: all clinical findings

Pair-wise scores calculated between DXplain (both ALL and REL) and ChatGPT, and between DXplain (both ALL and REL) and Gemini, with and without labs, were compared using mixed model ANOVA for repeated measures. The frequencies with which DXplain, ChatGPT and Gemini listed the correct diagnosis higher in their respective differentials were compared using logit-link generalized linear model for which the estimation was carried out by the generalized estimating equations (GEE) approach.

© 2025 Feldman MJ et al. *JAMA Network Open.*
