# Supplementary material for: Dedicated AI Expert System vs Generative AI With Large Language Model for Clinical Diagnoses
Source: JAMA Netw Open. 2025 May 29;8(5):e2512994. doi: 10.1001/jamanetworkopen.2025.12994 (PMC12123466; doi:10.1001/jamanetworkopen.2025.12994)
Supplement: Supplement 2. — Data Sharing Statement [file jamanetwopen-e2512994-s002.pdf]

Data Sharing Statement

Feldman. Dedicated AI Expert System and Generative AI With Large Language Model for Clinical Diagnoses. *JAMA Netw Open*. Published May 29, 2025.
doi:10.1001/jamanetworkopen.2025.12994

Data
Data available: Yes
Data types: Data (not involving human participants)
How to access data: Data (differential diagnoses of the LLMs and DXplain diagnostic decision support tool) available upon request to feldman.mitchell@mgh.harvard.edu
When available: With publication

Supporting Documents
Document types: None

Additional Information
Who can access the data: researchers whose proposed use of the data has been approved
Types of analyses: Studies on computer based diagnostic decision support
Mechanisms of data availability: after approval of a proposal
